# Conceptualization of Participation: A Qualitative Synthesis of Brain Injury Stakeholder Perspectives

**DOI:** 10.3389/fresc.2022.908615

**Published:** 2022-07-22

**Authors:** Caitlin Rajala, Camden Waterhouse, Emily Evans, Kimberly S. Erler, Michael J. Bergin, Sarah M. Bannon, Mary D. Slavin, Lewis E. Kazis

**Affiliations:** ^1^Department of Physical Medicine and Rehabilitation, Spaulding Rehabilitation Hospital, Harvard Medical School, Boston, MA, United States; ^2^School of Health and Rehabilitation Sciences, MGH Institute of Health Professions, Boston, MA, United States; ^3^Center for Health Outcomes and Interdisciplinary Research, Department of Psychiatry, Massachusetts General Hospital, Harvard Medical School, Boston, MA, United States; ^4^Rehabilitation Outcomes Center, Spaulding Rehabilitation Hospital, Boston University School of Public Health, Boston, MA, United States; ^5^Rehabilitation Outcomes Center, Spaulding Rehabilitation Hospital, Harvard Medical School and Boston University School of Public Health, Boston, MA, United States

**Keywords:** participation, stakeholder, brain injury, qualitative, acquired brain injury, scoping review, synthesis

## Abstract

**Background:**

The return to participation in meaningful life roles for persons with acquired brain injury (pwABI) is a goal shared by pwABI, their families, clinicians, and researchers. Synthesizing how pwABI define participation will help to identify the aspects of participation important to pwABI and can inform a person-centered approach to participation outcome assessment. To-date, the qualitative synthesis approach has been used to explore facilitators and barriers of participation post-stroke, and views about participation among individuals with stroke in the UK.

**Objectives:**

This paper's objectives are to (1) conduct a scoping review of qualitative literature that defines and characterizes participation from the perspective of pwABI of any type, (2) synthesize how pwABI define and categorize participation, and (3) link the themes identified in the qualitative synthesis to the International Classification of Functioning, Disability, and Health (ICF) using standardized linking rules to enhance the comparability of our findings to other types of health information, including standardized outcome measures.

**Methods:**

We completed a scoping review of qualitative literature. Our search included PubMed, APA PsychInfo, CINAHL, and Embase databases and included articles that (1) had qualitative methodology, (2) had a sample ≥50% pwABI, (3) had aims or research questions related to the meaning, definition, perception, or broader experience of participation, and (4) were in English. Qualitative findings were synthesized using Thomas and Harden's methodology and resultant themes were linked to ICF codes.

**Results:**

The search identified 2,670 articles with 2,580 articles excluded during initial screening. The remaining 90 article abstracts were screened, and 6 articles met the full inclusion criteria for the qualitative synthesis. Four analytical themes emerged: (1) Essential Elements of Participation (2) How pwABI Approach Participation, (3) Where pwABI Participate, and (4) Outcomes of Participation. Each overarching theme included multiple descriptive themes.

**Conclusion:**

In this paper, we identified themes that illustrate key components of participation to pwABI. Our results provide insight into the complex perspectives about participation among pwABI and illustrate aspects of participation that should hold elevated importance for clinicians and researchers supporting participation of pwABI.

## Introduction

Persons with acquired brain injury (pwABI), due to either stroke or trauma, can experience long term changes in participation across multiple facets of life (1–6). Acquired brain injury (ABI) has been found to result in a higher prevalence of disability when compared to the general population (7), and to result in long-term impacts on daily function (8). Helping pwABI engage in valued tasks and roles is a goal shared by pwABI, their families, clinicians, and researchers (9–11). The International Classification of Functioning, Disability and Health (ICF) terms this aspect of functioning, “participation” and defines it as “involvement in a life situation” (12, 13). Research aimed at improving participation post-ABI, however, has been hindered by well-recognized challenges in participation measurement, including acknowledged inconsistencies in how participation is defined (9, 11, 14–19).

Understanding how pwABI define and conceptualize participation is a critical part of measuring, and thereby improving, participation for pwABI. Qualitative research provides an avenue for direct insight into the perspectives of pwABI (9, 10, 20). Results from several qualitative studies related to participation have identified subjective and contextual elements of participation, such as satisfaction, agency, and environment as important to stakeholder groups (9–11, 19, 20). Synthesizing evidence from qualitative sources defining participation will help to identify the aspects of participation important to pwABI. Characterizing this information using a widely accepted framework can help inform a person-centered approach to participation outcome measurement and improve understanding of participation following ABI (18, 21–24).

Qualitative syntheses provide a systematic approach to compiling findings from multiple qualitative studies (25, 26) and may help to characterize how pwABI perceive participation. The qualitative synthesis approach has previously been used to explore facilitators and barriers of participation post-stroke (27), and to explore views about participation among individuals with stroke in the UK (10). The ICF, published by the World Health Organization in 2001, is the predominant biopsychosocial framework of health used in disability and rehabilitation research (12). The ICF is designed to provide a universal classification of health and disability and to create a common language to enhance comparability of healthcare information from multiple sources (12). Specific ICF linking rules have been established to help characterize health-related information from multiple sources (22). Earlier iterations of these rules have been used to link participation-related qualitative data and outcome metrics to the ICF (15, 23, 28), and to link themes from qualitative syntheses to the ICF conceptual model (29).

To extend this work, our objectives are to (1) conduct a scoping review of qualitative literature that defined or characterized participation from the perspective of pwABI of any type (i.e., not limited to individuals with stroke), (2) synthesize how pwABI define and categorize participation, and (3) link the themes identified in the qualitative synthesis to the ICF using standardized linking rules (22) to enhance the comparability of our findings to other types of health information, including standardized outcome measures.

## Methods

### Literature Review

Scoping reviews are conducted with the primary purpose of determining and summarizing the breadth of current literature and identifying existing gaps in the literature (30). We conducted a scoping review to identify key qualitative literature in which pwABI defined and conceptualized participation, and to synthesize existing findings independently and in relationship to the ICF. This scoping review used Arskey and O'Malley's (30) guidelines as a foundation.

#### Literature Search and Identification

This literature search and synthesis conformed with PRISMA Scoping Review guidelines (31). We conducted a scoping literature review from February 2021-March 2021 using PubMed, APA PsychInfo, CINAHL, and Embase databases. Database search terms and initial results are listed in Table 1. Article titles and keywords were reviewed to identify qualitative studies related to ABI and participation (CR). Reference lists from identified articles were also reviewed for additional publications (CR, CW). Abstracts and article content from remaining articles were reviewed by team members (CR, CW) to further determine eligibility based on the following criteria: the article (1) used qualitative methodology, (2) had a sample with ≥ 50% pwABI, (3) had at least one research question or study aim related to the meaning, definition, perception, or broader experience of participation, and (4) was published in English. In the event of uncertainty about inclusion criteria at any stage of the literature search, research team members (CR, CW, EE) met and discussed the articles in question to reach consensus. Once key articles were identified, team members independently assessed the article abstracts and content, recording compliance with each of the above inclusion criteria for final consensus.

**Table 1:** Databases and search terms.

**Database**	**Search terms**
PubMed	Participation AND brain injury AND qualitative Defining Participation AND brain injury AND qualitative People with brain injury perspectives on participation Participation, assessment, qualitative research, brain injury Brain injury AND participation perspectives Perspectives on participation people with brain injury
APA PsychInfo and CINAHL	Participation AND brain injury AND qualitative Participation in brain injury AND qualitative people with brain injury AND participation TI disablit* AND Participati* AND (qualitative OR Interview* OR focus group* OR content analysis) OR thematic analysis health AND TI meaning of participat* AND (qualitative OR Interview* OR focus group* OR content analysis OR thematic analysis) Disability AND TI meaning of participat* AND (qualitative OR Interview* OR focus group* OR content analysis OR thematic analysis) 10*(qualitative OR Interview* OR focus group* OR content analysis OR thematic analysis) Disabilit* AND TI experience of participat* AND (qualitative OR Interview* OR focus group* OR content analysis OR thematic analysis)
Embase	“brain injury”:ti AND participation:ti AND qualitative:ti,ab,kw AND “focus group*”:ti,ab,kw AND interview*:ti,ab,kw. “brain injury”:ti AND participation:ti AND qualitative:ti,ab,kw OR “focus group*”:ti,ab,kw OR interview*:ti,ab,kw OR thematic analysis OR content analysis

* *Signifies the truncation wildcard used in database searches*.

#### Quality Assessment

Each included article was independently assessed by two researchers (CR, CW) using the Critical Appraisal Skills Program (CASP) Qualitative Checklist (32). The CASP Qualitative checklist is a widely used tool in health-related qualitative synthesis (33–36). Both researchers independently completed a CASP assessment for each article, then met to compare and discuss any disagreements in classification. We did not impose cutoffs for exclusion due to the limited number of articles and the limited evidence around excluding articles based on quality assessment (25).

### Analysis and Synthesis

#### Inductive Coding & Synthesis

We used Thomas and Harden's (25) approach, which outlines a three-step process using thematic analysis, to guide our qualitative synthesis. Thematic analysis involves close reading of text to identify data-driven patterns that “become the categories for analysis” (37). Codes are developed inductively using the results sections from the first article, then transferred to the succeeding articles and new codes are added as needed (25). Reviewers then develop descriptive themes that are representative of groups of identified codes, and align closely to the literature being synthesized (25). Lastly, reviewers develop analytical themes, which require reviewers to apply their own interpretation of article findings (25).

Three researchers (CR, CW, EE) reviewed the articles, then independently coded the article results. The team then met to compare codes and establish broader descriptive themes. Two researchers (CR, CW) then independently reevaluated the coded text in relation to the descriptive themes, confirming consistency of the ascribed text and reaching a consensus in instances of disagreement. If the two researchers could not reach consensus, the third team member involved in coding was consulted to reach a resolution. Descriptive themes were organized into analytical themes through group discussion of the same three research team members.

#### ICF Linking

We linked the descriptive themes identified in the synthesis to the ICF model using standardized linking rules (22, 38, 39). We also compared the ICF categories identified in our linking process to the ICF Comprehensive Traumatic Brain Injury (TBI) and Stroke Core Sets (40, 41). Whereas, the full ICF Classification is intended to be universal, diagnosis-specific ICF Core Sets were developed to identify “essential categories from the full ICF classification” most relevant to specific health conditions (42). We compared the ICF categories identified through linking to the TBI and Stroke Core Sets to examine the alignment of our inductive findings with previously established “essential categories” (42). We referenced both the Comprehensive TBI and Stroke Core Sets for this analysis because ABI encompasses both diagnoses (40, 41).

The ICF linking rules follow a standardized process of identifying *main concepts* and *additional concepts* in specific health information, and then linking those with the ICF's hierarchically organized alphanumeric structure (22). If the information is beyond the scope of the ICF, or the information is not specific enough to link to a component of the ICF “Not covered (nc),” and “Not defined (nd),” are used, respectively. Otherwise, the health information is assigned to the component level (e.g., (e) Environmental Factors) when information was not specific enough to link to the first or chapter level (e.g., d4 “Mobility”) or the more precise ICF categories at the second, third, or fourth levels (e.g., d630 “Preparing meals”) (22). For this analysis, we also used this process when one chapter did not encompass the full meaning of the theme (22). Each ICF category also contains an “other specified” option when a concept is not represented by the specific categories at the second, third, or fourth levels, or an “unspecified” option to be used if a concept fits within a chapter but lacks information for a more precise ICF category (22). The linking rules also require that the perspective adopted in the linked information be identified (22). The linking process is detailed in Cieza et al. (22).

Three researchers (CR, CW, EE) used the ICF linking rules to link descriptive themes from the synthesis to the ICF (22). One of the researchers (EE) attended the English-language ICF workshop, which provided information on how to effectively link health information to the ICF (43), and two researchers (CR, CW) completed the recommended training modules through the ICF e-learning tool to familiarize themselves with the ICF (44). All researchers reviewed fundamental ICF resources and articles related to the ICF and the linking rules (12, 13, 22, 38, 39). Themes were first independently linked to the full ICF Classification by each researcher (45), then all three researchers compared their results for consistency and resolved discrepancies through group discussion. It was determined a priori that linking would only be done to the second level of the ICF (15, 22, 46). The ICF categories linked to the main concepts of the descriptive themes were then checked against the TBI and Stroke Core Sets to determine if they were included (40, 41).

## Results

### Summary of the Literature Search

**Figure 1:**
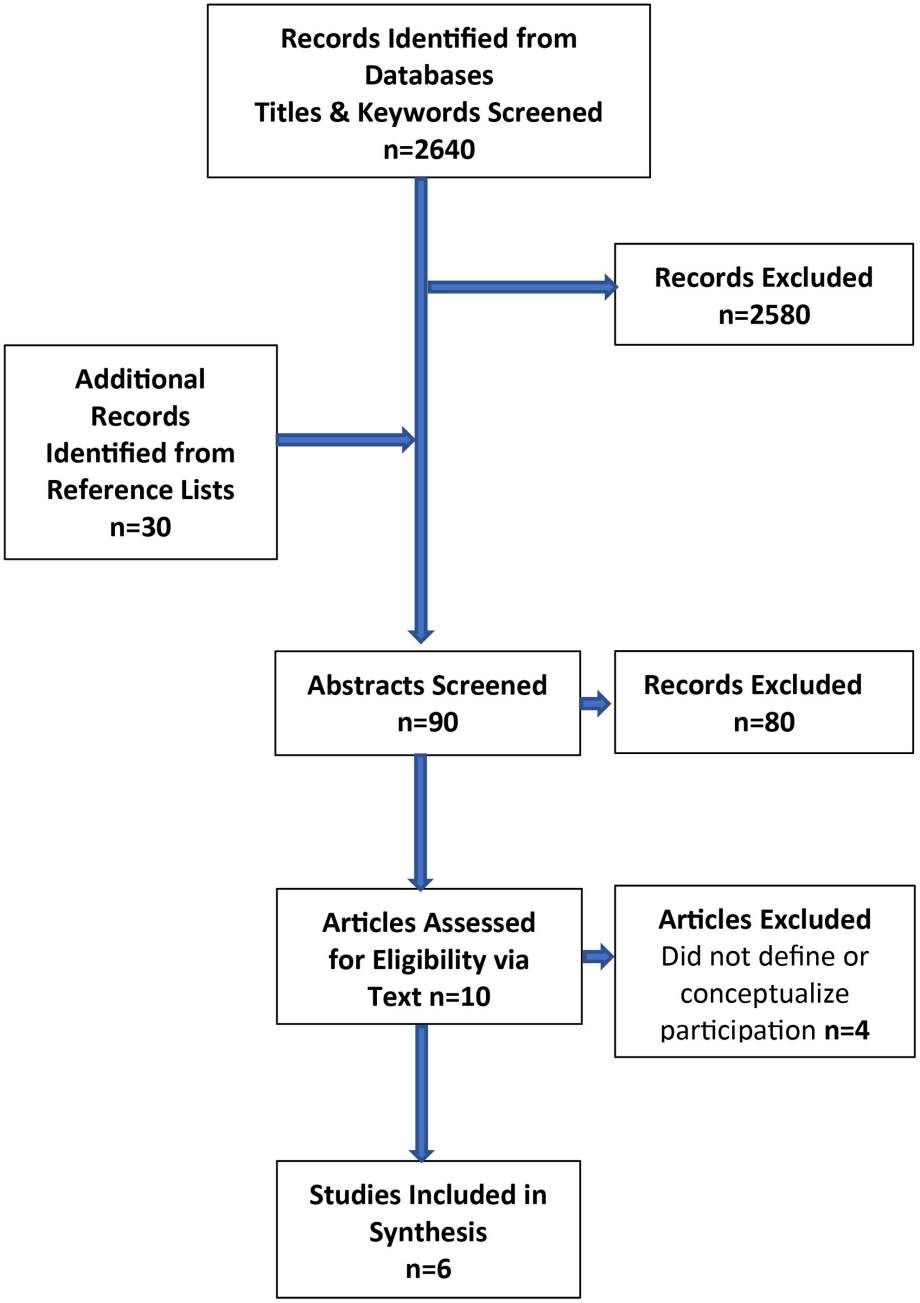
Flow chart of article selection process.

The titles and keywords of 2,670 articles were initially screened, and 2,580 articles were excluded. Ninety qualitative articles related to participation and ABI were identified and abstracts were screened. Ten articles were identified for the final vetting process outlined above. Six articles met the full inclusion criteria for the qualitative synthesis (Figure 1).

### Article Characteristics and Quality Assessment

Six articles were included in the synthesis (47–52) (Table 2). One article used mixed-method design (50), and one drew on a subset of data from a larger qualitative study looking at stakeholders' perspectives on participation in persons with disabilities (48). Another article published its methods (53) separately from its results (49). Theoretical foundations of articles included grounded theory (48, 49, 53) and phenomenology (51), with other articles citing explanatory sequential design (50) and exploratory design (52). One article stated that their qualitative design “emerged gradually” over the course of the research (47). All articles used either interviews (47, 49, 51–53) or focus groups (47, 48, 50) for data collection purposes. One study used photovoice (50), another included working groups as part of their process (47), and one outlined the use of “total communication technique” in their interviews and focus groups, which allowed for participants with speech difficulty to participate with gestures or writing (51). The total number of participants included in the articles was 176, ranging from 11 participants (52) to 63 participants (48) per study.

**Table 2:** Article and sample characteristics.

**Article**	**Sample characteristics**	**Stakeholder type**	**Data collection methods**	**Country and environment**
Amarshi et al. (49, 53)	Total *n =* 12 Reported Male: 58.3% Race: NR Ethnicity: 75% White, 16.7% Asian, 8.3% African American Age: 60+ Injury type: Stroke Time since injury: 1–9 years	Person w/ABI	Structured Interviews	Canada; Living at home
Barclay-Goddard et al. (50)	Total *n =* 16 Reported Male: 75% Race: NR Ethnicity: NR Age: 44–77 Injury type: Stroke Time since injury: mean 3.8 years	Person w/ABI	Focus Groups and Photovoice	Canada; Living in the community
Schipper et al. (47)	Total *n =* 62 Reported Male: 46.8% Race: NR Ethnicity: NR Age: 27–60 Injury type: Stroke, TBI, Other* Time since injury: minimum of 1 year	Mixed Injury Population	Interviews, Focus Groups, and a Working Group	Netherlands; Living on their own or with family
Haggstrom et al. (52)	Total *n =* 11 Reported Male: 45.5% Race: NR Ethnicity: NR Age: 38–62; mean 55 Injury type: Stroke and Mod. TBI Time since injury: 3–6 years	Person w/ABI	Open-ended Interviews	Sweden; NR
Fryer et al. (51)	Total *n =* 12 Reported Male: NR Race: NR Ethnicity: 66.7% White British, 16.7% Pakistani, 8.3% White German, 8.3% British Pakistani Age: 16–68 Injury type: Stroke Time since injury: NR	Person w/ABI; Caregiver	Semi-Structured Interviews	England; NR
Hammel et al. (48)	Total *n =* 63, *n =* 56 reported demographic information Reported Male: 55.4% Race: 38% White, 49% Black or African American, 2% Native American, 2% Asian, 9% Other Ethnicity: 9% Hispanic or Latino Age: 18–70+ Injury type: Stroke, TBI, Other** Time since injury: NR	Mixed Injury Population	Focus Groups	USA; Single family home, apartment, supervised group living, and other settings

* *Other: includes brain tumor, infection, out-of-hospital cardiac arrest, and unknown*.

** *Other: includes spinal cord injury and other*.

We evaluated all studies using the CASP Qualitative Checklist (32). All articles were determined to be of moderate to good quality, but some only reported “ethical approval” rather than “ethical practices” (48, 52), or did not specify use of triangulation (51) or data saturation (48, 52). Most had limited information about contradictory data (47–49, 51, 52) and assessment of researcher bias (47, 51, 52). One study did not report formal ethical approval, but oversight by a steering committee (47). Full details of the CASP checklist are provided in Table 3.

**Table 3:** CASP Quality appraisal for all articles.

**CASP Questions**	**Amarshi et al. (49, 53)**	**Barclay-Goddard et al. (50)**	**Fryer et al. (51)**	**Haggstrom et al. (52)**	**Hammel et al. (48)**	**Schipper et al. (47)**
1. Was there a clear statement of the aims of the research?	Y	Y	Y	Y	Y	Y
2. Is a qualitative methodology appropriate?	Y	Y	Y	Y	Y	Y
3. Was the research design appropriate to address the aims of the research?	Y	Y	Y	CT	Y	Y
4. Was the recruitment strategy appropriate to the aims of the research?	Y	CT	CT	CT	Y	Y
5. Was the data collected in a way that addressed the research issue?	Y	Y	Y	Y	Y	Y
6. Has the relationship between researcher and participants been adequately considered?	CT	CT	N	CT	Y	Y
7. Have ethical issues been taken into consideration?	Y	Y	Y	CT	CT	CT
8. Was the data analysis sufficiently rigorous?	Y	Y	Y	Y	Y	Y
9. Is there a clear statement of findings?	Y	Y	Y	Y	Y	Y
10. How valuable is the research?	Y	Y	Y	Y	Y	Y

*N, No; Y, Yes; CT, Can't tell (32)*.

### Participant Characteristics

Participant age ranged from 16 to (over) 70 years (47–53) (Table 2). Exact age parameters were not always explicit. Across the studies 25–53.2% of participants were of the female sex (47, 48, 50, 53). Of the one study that reported gender, 54.5% of the sample were women (52). Individuals identified as White were the largest racial group in the majority of articles, comprising 38–75% of the reported samples. Additional racial groups represented included African American, Black, Asian, Native American, and “other” (48, 49, 53). Ethnicity was reported in half of the studies (48, 49, 51, 53). Our inclusion criteria allowed for studies with a sample that included non-ABI populations if individuals with ABI comprised at least 50% of the sample (47, 48).

### Main Findings

#### Primary Synthesis

**Figure 2:**
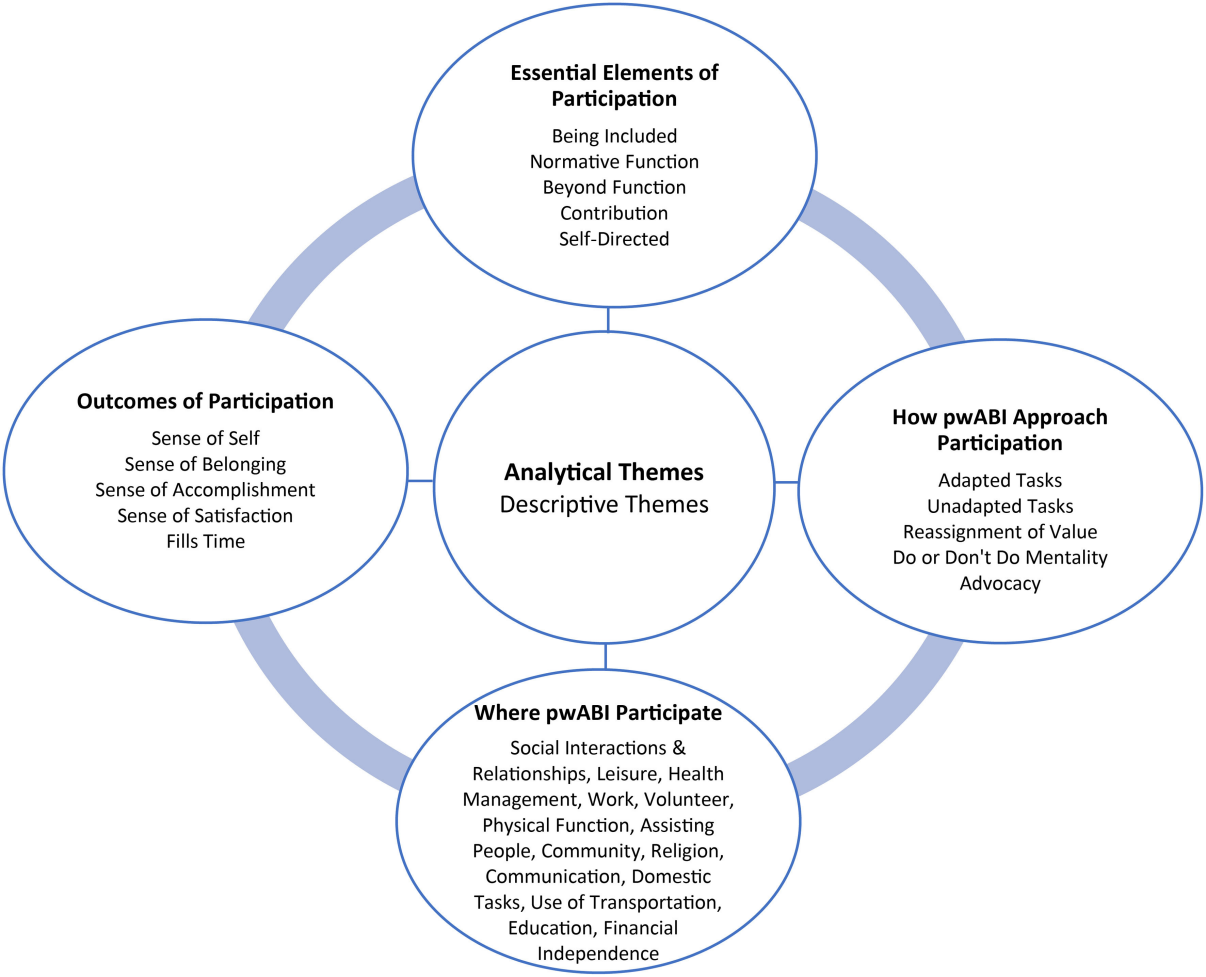
Analytical and descriptive themes.

This synthesis aimed to explore patterns across qualitative studies that outline how pwABI conceptualize participation. Four analytical themes emerged during the synthesis (1) Essential Elements of Participation, (2) How pwABI Approach Participation, (3) Where pwABI Participate, and (4) Outcomes of Participation. Each of these overarching themes included multiple descriptive themes from the articles which will be outlined in the following subsections (Figure 2 and Table 4).

**Table 4:** Examples of article quotes that support each theme.

**Analytical theme**	**Descriptive theme**	**Examples of quotes**
Essential Elements of Participation	Being Included	“It means taking part, being one of the team...a cog within a wheel” The stroke survivors described how participation meant being part of something, which involved working in co-operation with others.” (51)
	Beyond Function	“Participation can be partial, from the perspective of society, but full and meaningful in the eyes of the person it involves.” (47)
	Normative Function	“Being able to do favorite activities alone which had always been done alone, was described as a form of participation…” (51)
	Contribution	“In resistance to popular perceptions about people with disabilities as perpetually receiving help, the root of participation for many participants was defined not by what they can get from other people, but instead by what they can contribute back to others.” (48)
	Self-Directed	“For example, participants defined participation in life as: Just to be able to do whatever you want to do to your fullest extent to the best of your ability.” (48)
How pwABI Approach Participation	Adapted Tasks	“This is a one handed knitting holder. Someone in the group suggested I try it out so I would be able to knit. It's a good thing that I can use it.” (50)
	Unadapted Tasks	“Let's say I'm watching soaps...Discovery Channel...! turn the channel, or watch a different programme because I can't remember the stories. Because the memory is not there. And likewise too for reading, I don't read much. Because I can't remember.” (49)
	Reassignment of Value	“The informants also revalued the “few” things they did for others because these things and the people concerned had become much more significant to them, making these activities more important for their sense of participation than they had been before.” (52)
	Advocacy	“We have a unique insight into life that a lot of people don't have. I think that's really important when we're talking about participation that we can share with others that haven't experienced this... from our perspective.” (48)
	Do or Don't Do Mentality	“You can actually do more, than you thought you would dare, so you just have to do it.” (47)
Where pwABI Participate**	Social Interactions & Relationships	“The informants' experiences reflected how their participation was enhanced by prioritization of activities conducted with those who made them feel good, such as people close to them, persons who had their own experiences of having a disability and pets.” (52)
	Leisure	“Well after I had my stroke, it was about 2 months after I went back to the [gym].” (50)
Outcomes of Participation	Fills Time	“Say if you did crosswords or Sudoku and that takes away hours of your time. I mean that's how I spend my time…it keeps me active mentally.” (50)
	Sense of Accomplishment	“I can still do the things that I did, maybe in a different way, but I can still accomplish this or that.” (48)
	Sense of Belonging	“Other stroke survivors described the benefits of socializing, such as maintaining old and developing new friendships, being able to relate to others with similar experiences and the enjoyment of having companionship while participating in their leisure activities.” (49)
	Sense of Satisfaction	“Doing these tasks, taking care for others, gives me a sense of worth and satisfaction.” (47)
	Sense of Self	“It was apparent that participation had a profound meaning for stroke survivors, that of defining who they are. “Doing” and “being” were often mentioned simultaneously, suggesting that they are intertwined.” (51)

** *There were additional components of the analytical theme “Where pwABI Participate,” which include the descriptive themes of Work, Volunteer, Education, Health Management, Community, Assisting People, Domestic Tasks, Religion, Financial Independence, Communication, Physical Function, and Use of Transportation. Additional examples of these components can be viewed in Supplemental Table 1*.

##### Essential Elements of Participation

The analytical theme, Essential Elements of Participation, represents the factors identified by participants as intrinsic to participation (47–52). We categorized these references into 5 descriptive themes: (1) Being Included, (2) Beyond Function, (3) Normative Function, (4) Contribution, and (5) Self-Directed (47–52).

Being Included characterizes how being valued, respected, and treated as an equal member of society is a defining aspect of participation for pwABI (47–52). One participant stated, “To participate fully in life is to interact physically and mentally and socially with your peers and others in the community at large to the extent that you can” (48). Participants spoke about being able to be oneself and be accepted (51, 52), but also articulated alternative experiences where their value as a member of society, or to those around them, was no longer recognized (47, 50, 51). “…I've lost quite a bit of my stature,” one participant stated, “and um now I'm just being treated like as if I'm a necessary evil” (50).

Hammel et al. (48) also highlighted the duality with which some participants both emphasized societal inclusion as integral to participation and emphasized a need to retain an identity independent of general society. This tension between disability identity and a broader societal identity is prevalent throughout the literature in this synthesis (48). This contrast was also illustrated in two other descriptive themes in this category, Beyond Function and Normative Function (47, 48, 50–52). Some pwABI appeared to conceptualize participation as something outside the normative standards often used to classify participation, such as how much or how often they participate, or level of independent function (47, 48, 50–52). Others alternatively considered participation to be synonymous with normative standards (47, 48, 50–52). These represented two distinct, and often contradictory, interpretations of how pwABI characterized participation.

There were several examples in the text that encompassed perspectives about new iterations of participation. For example, Hammel et al. wrote:

Many participants distinguished engagement from functionally independent performance. As one participant summarized, “I don”t want to be restricted by function'. Rather, it involved “freedom to pursue happiness, pursue whatever you want to do”. This pursuit involved going beyond an exclusive focus on day to day survival, to participation in opportunities that were highly meaningful, fun, enriching, and/or satisfying (48).

Schipper et al. (47) similarly summarized participant perspectives, stating, “… quality of participation is more important than the degree of participation…” Alternatively, some participants defined participation as something that required a return to a preinjury self, or an ability to function according to generalized standards. For example, Hammel et al. (48) wrote, “…remediation of impairment was viewed as a prerequisite to full participation.” Participant statements also included references to a normative point of view, a lens through which participation was classified: “I wanted to be the old me. I resisted and wanted to be normal again!” (47). The differences in these perspectives could be viewed as reflective of different stages of the recovery process, as noted by Schipper et al. (47). It is arguably also reflective of the diverse nature of ABI and evidence that overly simplistic approaches to conceptualizing participation may fail to capture critical, person-centered areas of focus.

Another element identified as a defining characteristic of participation in several of the studies (48, 51, 52), and an important element of participation in remaining studies (47, 49, 50) was that of participant preference and choice. This was embodied in the synthesis theme, Self-Directed, which was defined as choice and control being integral to participation for pwABI. Authors noted the role of preference in participants' classification of participation (47–52), the importance of options and access (48, 51, 52), and highlighted the diversity and range of participant preferences (48, 50–52). Participants equated meaning with interest and self-determination, and ultimately with participation as a whole (47, 49–52). Statements such as “I think that it is very important for me… to be involved in decision-making… even if I can't manage to do the activity by myself” (52), and “…. What I care about is what I want to do…” (48), illustrate these points. Fryer et al. (51) noted the variety of types of participation listed by participants, and all articles noted that people either defined or measured their participation in relation to their investment in and ability to self-determine (47–52).

Contribution was the final descriptive theme grouped beneath the analytical theme, Essential Elements of Participation. Though contribution was sometimes referenced in articles as a type of activity, this theme was applied to text that classified contribution as a defining characteristic of participation (47–49, 52). Schipper et al. (47) wrote, “…This sense of fullness is enhanced when people feel engaged and if they can contribute to society or a larger whole. Participation is thus about taking part, giving something and being someone in a specific context…” Hammel et al. (48) echoed this, stating “…the root of participation for many participants was defined not by what they can get from other people, but instead by what they can contribute back to others.” Participants also spoke about contribution in parallel with Being Included, as a way of being valued and seen in society (47, 48, 52). One participant stated, “…it's having an access and opportunity to make a contribution and to give of oneself I think. That's what fully participating means to me” (48).

##### How PwABI Approach Participation

Each of the articles in the synthesis contained participant perspectives that reflected the diverse ways in which people approach the act of participation (47–52), a theme termed How pwABI Approach Participation. Five descriptive themes were included under this analytical theme: (1) Adapted Tasks, (2) Unadapted Tasks, (3) Advocacy, (4) Reassignment of Value, and (5) Do or Don't Do Mentality.

The theme of Adapted Tasks emphasized findings related to participants' approach to participation post-injury in one of two ways: by adapting their previous activities as necessary (49, 52) or by refocusing their participation on new activities (47–50, 52). For example, several participants made adaptations to their former activities post-ABI, such as learning to play golf with one hand (49) or accepting functional differences in order to continue to participate in spin classes (52). Alternatively, other statements from participants highlighted that some of them continued to participate by finding new activities to replace those that they could no longer perform, or to continue to access domains of participation they lost conventional access to post-ABI. Haggstrom et al. (52) wrote, “… they were trying to enhance their participation by taking on more voluntary work than they had done before...” and “Yet another strategy was to get involved in new social contexts by establishing new relations.”

Another descriptive theme, Unadapted Tasks, emerged to capture instances in which participants did not adapt their methods of participating. Participants instead articulated experiences of discontinuing participation as a result of their ABI (47, 49–52), or of continuing with activities in an unchanged way (47–52). Participants communicated a cessation of activities because of cognitive challenges (49), fear or lack of confidence (49, 51), and physical limitations (49, 50). For example, one participant stated, “Well, I used to curl. I can't curl. I'm afraid to fall on the ice” (49). Alternatively, some participants spoke about resuming activities they had done previously (49, 50), “‘…for me I joined a football pool again this year. I have no real interest in it… I'm just going because I've been doing it for 15 years…” (50). While the theme Unadapted Tasks represents two ends of the participation spectrum, it highlights an alternative to Adapted Tasks that many participants referenced.

The Advocacy theme was defined as influencing, enhancing, or promoting rights and perspectives for others or oneself (47, 48, 52). Some articles highlighted participants' desire to share insights they gained from having an ABI, or noted that networks of people with disabilities allowed for an increased ability to participate (48). Participants expressed a desire to influence society through political or organizational means (47, 52), and self-advocacy was also a component of this theme (48, 52).

Some participants also spoke about how their injuries caused them to ascribe new meaning or value to aspects of participation, a theme we defined as Reassignment of Value (47, 52). Post-injury, participants expressed a shift in how important certain people or activities were (47, 52) and, as Haggstrom et al. (52) noted, how important things were “for their sense of participation.” One participant stated:

For me, it's now enough to be a good mother and wife. I'm able to clean the house, wash the children and give them all clean clothes. Doing these tasks, taking care for others, gives me a sense of worth and satisfaction (47).

Lastly, How pwABI Approach Participation included a descriptive theme, Do or Don't Do Mentality (47–51). While this theme did not outline a task-specific approach pwABI took in relation to participation, it captures an important element reflected in the papers. Some participants approached participation with a philosophy grounded in doing and pushing (47–49, 51), while other participants emphasized a loss or withdrawal (49, 51). One person stated, “I don't think there's ever been a time up until this thing, where I would ever walk away from something…that side of me has totally gone” (51). As with the theme Essential Elements of Participation, How pwABI Approach Participation highlights the varied ways in which pwABI address participation, and the range of approaches through which they enact participation post-injury.

##### Where PwABI Participate

In all the articles selected for the synthesis, participants spoke about key domains in which participation did, or should, occur (47–52). References to Social Interactions and Relationships were most common and were mentioned multiple times in all articles (47–52), whereas references to leisure activities or Leisure in general were the second most common (47–52). Additionally, Health Management (references to actively maintaining health and wellbeing) (47–51), Community (47–49, 51, 52), Physical Function (49–51), Use of Transportation (references to driving or using public transportation) (48–50), Assisting People (47, 51, 52), Work (47–49, 51), and Volunteer (48–50) were mentioned within multiple articles. Less frequently mentioned were Domestic Tasks (47, 48), Communication (47, 51), Education (48, 49, 51), Religion (48), and Financial Independence (48). These themes serve to locate the more conceptual aspects of participation highlighted in the analytical themes above, illustrating the importance of social interaction and relationships in participation for pwABI.

##### Outcomes of Participation

In each of the articles, references to the Outcomes of Participation, the positive or negative experiences that resulted directly from an act of participation, were discussed (47–52). We classified these as (1) Sense of Belonging, (2) Sense of Self, (3) Sense of Accomplishment, (4) Sense of Satisfaction, and (5) Fills Time. Sense of Belonging shared some similarities to Being Included, however the former is a product of participating while the latter is a prerequisite. One participant noted, “…when we go into the meetings, we can share the frustration. Um, and encourage each other. Its very important” (49) while another stated, “I guess I joined it for the social connection” (50). Alternatively, some participants referenced feeling excluded as a result of trying to participate (49, 52).

Three of the articles contained text related to feelings of accomplishment as a byproduct of participation (48–50). In Barclay-Goddard et al. (50), Sense of Accomplishment was represented by pictures of activities participants felt achievement over completing, for example, learning to knit or make art in an adapted way, or managing a train trip with a spouse. Additionally, participants referenced accomplishment more broadly, and the act of participation as a way in which they were able to illustrate their ability to accomplish things (48, 51).

Sense of Self was a theme present in four of the articles (47–49, 51). This theme represented text where participants or authors spoke about the ways in which participants' identities were impacted by their participation. As Amarshi et al. (49) wrote, “…These activities represented the stroke survivor's self-identity and provided purpose to their lives.” References to a Sense of Self in the articles reflected that pwABI often align their identity with their ability to participate in personally meaningful roles or activities and experience feelings of purpose, worth, and confidence as a result of said participation (47–49).

The themes Fills Time and Sense of Satisfaction were less prevalent in the articles, but still reflected in the text as a way in which pwABI experienced the results of participation. Fills Time was represented in two articles (49, 50) and Sense of Satisfaction was reprsented in three (47, 50, 52). Fills Time captured text in which participants noted participation as “something to do,” (50) or a way to “occupy their time” (49). Text represented under Sense of Satisfaction included statements about how participants felt satisfaction as a result of fulfilling roles (47, 50), but also in relation to feeling satisfaction with performance and participation as a whole (50, 52). It is important to note that while these were the themes seen consistently across papers in reference to Outcomes of Participation, they do not represent all outcomes of participation experienced by pwABI.

#### ICF Linking

Results from the linking of the descriptive themes to the ICF are detailed in Tables 5–**8**. Definitions for each ICF alphanumeric code can be found in the publicly available ICF Browser (45). Per the ICF linking rules, the *main concept* of each descriptive theme was identified and *additional concepts* were identified when appropriate (22). Several of the descriptive themes from the synthesis had *main concepts* that we linked broadly to an ICF component (e.g., (d) Activities and Participation) (Tables 5, 6). The themes Beyond Function, Normative Function, Adapted Tasks, and Unadapted Tasks all had *main concepts* that were linked to component (d) Activities and Participation since they could encompass all types of activities and participation and could not be linked to a more specific category (Tables 5, 6). Reassignment of Value had an *additional concept* that was also linked to component (d). Both Adapted Tasks and Unadapted Tasks had an *additional concept* that was linked to component (e) Environmental Factors as all environmental domains could contribute to adapting tasks or serve as a barrier to participation, but the *main concept* did not include specific environmental factors that could be linked to a specific category (Table 6).

**Table 5:** ICF linking to themes from essential elements of participation.

**Descriptive theme**	**Perspective adopted in information**	**Main concept**	**Additional concepts**	**ICF category of main concept (2nd level)**	**ICF category of additional concepts**	**MC in TBI core set**	**MC in stroke core set**
Beyond Function	Appraisal	It doesn't matter what society thinks participation is, it matters what I think participation is.	N/A	d (all)	N/A	Y*	Y*
Normative Function	Descriptive; Capacity	Participation is what you did and how you did things before your injury, and what “everybody else does”	N/A	d (all)	N/A	Y*	Y*
Contribution	Descriptive; Performance	Making a contribution on an individual/societal level is essential to participation	N/A	d898**- making a contribution; d998**-making a contribution; d798**- making a contribution; d698**- making a contribution; d179**- making a contribution; d230	N/A	Y (d230 only)	Y (d230 only)
Self-Directed	Appraisal	Having choice and control is essential to participation	Making your own decisions	d940	d177	N	N
Being Included	Appraisal	Being viewed by society as valuable and equal is essential to participation	Being supported by those I interact with	e499***	e399***	N	N

MC, Main concept; Y, Yes; N, No; Y*, Chapter is referenced in the Core Set, but not all chapter codes are included.

** *Codes are other specified*;

*** *Codes are unspecified*.

**Table 6:** ICF linking to themes from how pwABI approach participation.

**Descriptive theme**	**Perspective adopted in information**	**Main concept**	**Additional concepts**	**ICF category of main concept (2nd level)**	**ICF category of additional concepts**	**MC in TBI core set**	**MC in stroke core set**
Adapted Tasks	Descriptive; Performance	Changing how you do an activity or the types of activities you do to align with post-injury abilities.	Tools and supports you utilize to adapt participation; a personal approach to participation	d (all)	e (all); pf	Y*	Y*
Unadapted Tasks	Descriptive; Capacity	Participating the same way you did pre-injury or not at all	Not using tools and supports to adapt participation; a personal approach to participation	d (all)	e (all); pf	Y*	Y*
Do or Don't Do Mentality	Descriptive; Performance	Responses to post-injury participation challenges	N/A	pf	N/A	Y	Y
Reassignment of Value	Appraisal	A change in the way you value, or assign importance to, activities after injury	All types of activities and participation	pf	d (all)	Y	Y
Advocacy	Descriptive; Performance	Promoting rights and perspectives of self and/or others	Human rights and political advocacy; communicating needs to, or for, other people	d998**- advocacy	d940; d798**-self-advocating and advocating for others in interpersonal relationships; d950	N	N

MC, Main concept; Y, Yes; N, No; Y*, Chapter is referenced in the Core Set, but not all chapter codes are included.

** *Codes are other specified*.

The descriptive themes Being included, Social Interactions and Relationships, Education, Community, Communication, Physical Function, Use of Transportation, Domestic Tasks, and Sense of Belonging all had *main* or *additional concepts* linked to “unspecified” categories (Tables 5, 7, 8). The *main concepts* of the descriptive themes Contribution and Advocacy were linked to the “other specified” categories, to articulate concepts not otherwise represented in the ICF chapters (Tables 5, 6). The *additional concepts* of the descriptive themes Advocacy, Health Management, and Social Interactions and Relationships were also linked using the “other specified” categories (Tables 6, 7).

**Table 7:** ICF linking to themes from where pwABI participate.

**Descriptive theme**	**Perspective adopted in information**	**Main concept**	**Additional concepts**	**ICF category of main concept (2nd level)**	**ICF category of additional concepts**	**MC in TBI core set**	**MC in stroke core set**
Social Interactions and Relationships	Descriptive; Performance	Interacting and having relationships with others	Socializing; need for people	d799**	d920; d798*- the need for interaction	N	N
Leisure	Descriptive; Performance	Things you do for enjoyment	N/A	d920	N/A	Y	Y
Work	Descriptive; Performance	Paid employment	N/A	d850; d845	N/A	Y	Y
Volunteer	Descriptive; Performance	Unpaid work	N/A	d855	N/A	Y	Y
Community	Descriptive; Performance	Involvement in community activities	N/A	d999**	N/A	N	N
Health Management	Descriptive; Performance	Managing your health and wellbeing	Seeking out health expertise and treatment	d570	d598*- seeking out health expertise and treatment	Y	Y
Education	Descriptive; Performance	Any form of education	N/A	d839**	N/A	N	N
Assisting People	Descriptive; Performance	Helping other people	N/A	d660	N/A	Y	N
Communication	Descriptive; Performance	Communicating with others	N/A	d399**	N/A	N	N
Physical Function	Descriptive; Performance	The ability to move around	N/A	d499**	N/A	N	N
Use of Transportation	Descriptive; Performance	Using any form of transportation	N/A	d489**	N/A	N	N
Religion	Descriptive; Performance	Engagement in religious and/or spiritual activities	N/A	d930	N/A	Y	N
Domestic Tasks	Descriptive; Performance	Household maintenance	Household chores	d650	d649**	N	N
Financial Independence	Descriptive; Performance	Managing finances and being able to financially support yourself	Purchasing items and keeping track of money	d870	d620; d860; d865	Y	Y

*MC, Main concept; Y, Yes; N, No;*

* *Codes are other specified*;

** *Codes are unspecified*.

Two descriptive themes had *main concepts* that were linked to more than one ICF category (Tables 5, 7). Work was linked to both d850 and d845 since both ICF categories are directly related to paid employment. Contribution also had multiple ICF categories linked to the *main concept*, these included d230 and several “other specified—making a contribution” links within specific chapters (Table 5). The team considered linking Contribution to d660 “Assisting others” but agreed that the *main concept* of “Making a contribution on an individual/societal level” encompassed more than the act of assisting described in d660. Contribution was therefore linked to all modes of activities and participation within which contribution can occur.

The descriptive theme, Filling Time, was the only theme designated as “not covered” since the ICF did not have a category applicable to participation being a way to fill time. Several descriptive themes were linked to Personal factors (pf) since they represented references to personal approaches, responses, and feelings related to participation. Adapted Tasks and Unadapted Tasks both had *additional concepts* that were linked to (pf) since whether a person approached participation with an adaptive or nonadaptive framework depended to some degree on personal characteristics (Table 6). Do or Don't Do Mentality, Reassignment of Value, Sense of Accomplishment, Sense of Belonging, Sense of Satisfaction, and Sense of Self all had *main concepts* that were linked to (pf) since they were more reflective of types of responses and perspectives people had about participating or reflected feelings and emotional outcomes experienced because of participation (Tables 6, 8).

**Table 8:** ICF linking to themes from outcomes of participation.

**Descriptive theme**	**Perspective adopted in information**	**Main concept**	**Additional concepts**	**ICF category of main concept (2nd level)**	**ICF category of additional concepts**	**MC in TBI core set**	**MC in stroke core set**
Fills Time	Appraisal	Participating gives people a way to fill time	Daily activities	nc-filling time	d230	N	N
Sense of Accomplishment	Appraisal	Participating makes people feel accomplished	N/A	pf	N/A	Y	Y
Sense of Belonging	Appraisal	Participating makes you feel like you belong	Social support; being understood	pf	e399**; e499**	Y	Y
Sense of Satisfaction	Appraisal	Participating makes you feel satisfied	N/A	pf	N/A	Y	Y
Sense of Self	Appraisal	Participating strengthens your identity	N/A	pf	N/A	Y	Y

*MC, Main concept; Y, Yes; N, No;*

** *Codes are unspecified*.

The perspectives adopted in our descriptive themes included the “Appraisal” perspective and “Descriptive perspectives: Performance and Capacity” (22). For the analytical theme Where pwABI Participate, all descriptive themes were linked to the “Performance” perspective (Table 7), whereas all the descriptive themes in the analytical theme Outcomes of Participation were linked to the “Appraisal” perspective (Table 8). The analytical themes of Essential Elements of Participation and How pwABI Approach Participation had more varied perspectives, with descriptive themes linked to all three perspectives listed above (Tables 5, 6). Linking descriptive themes rather than items from a measure, or more granular qualitative data, presented some challenges, and the nuances of said themes were at times partially captured by the perspectives designated during the linking process. This was the case with the descriptive themes Normative Function and Unadapted Tasks, which were both linked to the “Capacity” perspective (Tables 5, 6). The “Capacity” perspective in the ICF “reflects the environmentally-adjusted ability of the individual in a specified domain” (12) and was therefore used in reference to themes that focused on functional and comparative ability over the realities of performance. Since Unadapted Tasks also referenced what people were or were not doing in their actual environment, the “Performance” perspective could also have been applicable, but it did not as accurately reflect the core meaning of the theme. Similarly, the “Appraisal” perspective added additional insight for themes with definitions that encompassed personal preference, choice, or satisfaction but that were not strictly linked to (pf): Beyond Function, Self-Directed, Being Included, and Fills Time (Tables 5, 8).

When the *main concepts* linked to the ICF were compared to the TBI and Stroke Core Sets there were several instances in which the categories that were linked were not present in the Core Sets (40, 41) (Tables 5–8). The Core Sets contain all (d) chapters, but do not contain some of the specific categories from the (d) chapters that could technically be included under the “d (all)” linking in our results. The Stroke and TBI Core Sets also do not contain “other specified” or “unspecified” categories. This is arguably because the Core Sets are intended to represent the “essential categories from the full ICF” and therefore categories that allow for additional specifications would not be included (42). Several of our *main concepts*, however, were linked to “other specified” or “unspecified” categories, indicating that there may be aspects of participation pwABI consider to be important that do not easily fit within current ICF Core Sets for Stroke or TBI. Specifically, the Stroke Core Set did not contain d660 or d930, which were linked to the descriptive themes Assisting People and Religion, respectively, though the latter was only mentioned in one of the articles (40, 41). Neither of the Core Sets contain d650, which was linked to the *main concept* for the theme Domestic Tasks (40, 41). Additionally, d940, which was linked to the *main concept* of our Self-Directed theme, is not included in either the TBI or Stroke Core Set (40, 41).

## Discussion

This paper aimed to (1) conduct a scoping review of qualitative literature that defined and characterized participation from the perspective of pwABI of any type, (2) synthesize how pwABI define and categorize participation and (3) link the themes identified in the qualitative synthesis to the ICF using standardized linking rules. Four analytical themes emerged from the existing literature: (1) Essential Elements of Participation, 2) How pwABI Approach Participation, 3) Where pwABI Participate, and (4) Outcomes of Participation. These themes and their related subthemes discuss participation in both conceptual and literal terms.

Rather than exploring what influences participation (e.g. barriers and facilitators), this paper aimed to draw from the small body of existing qualitative literature in which pwABI highlight the concepts they consider critical to participation. This review focused on article content that explored the underlying constructs that define participation rather than the variables that affect it. Not surprisingly, environment often played a key role, and participants classified environment as an important determinant of participation in all of the articles reviewed (47–52). Environment is known to heavily influence participation (9, 54–56), and many researchers have discussed the challenges of distinguishing between environment and participation in the ICF (18, 54, 55). Some leaders in the participation measurement field have also argued that while the intersection of environment and participation is important, conflating environment as part of participation, as opposed to an impacting force on participation, has contributed to flawed measurement of both participation and environment (18). Therefore, while environmental factors were included in themes when they were discussed as part of what defined participation, references to environmental factors that were mentioned purely as barriers and facilitators of participation were not included.

The descriptive theme most representative of the challenging intersection between environment and participation is the theme, Being Included. As noted above, this theme represents how being valued, respected, and treated as an equal member of society is integral to participation. This theme's relationship to society illustrates how some characteristics that pwABI consider integral to participation intersect with the environment. This aligns with Heinemann et al.'s work on the concept of enfranchisement (54, 57), which included one of the studies featured in this synthesis (48). Enfranchisement is a multidimensional construct that represents a person's perception of how their community values and supports their participation, and a person's resultant ability to participate according to their personal preferences (54, 57). Societal inclusion was emphasized as a defining component of participation throughout the literature in this synthesis (49–52).

The findings from this study are mirrored in other qualitative studies and syntheses of both ABI and other populations (9–11, 20). Our theme, Self-Directed, was reflected in articles by Van de Velde et al. (20), Martin Ginis et al. (11), and Woodman et al. (10). Van de Velde et al. (20) found that individuals with spinal cord injury highly valued their ability to make choices about their activities (20). Though several of the articles in our synthesis related meaningfulness of activities to choice (49–52), participants in Van de Velde et al. (20) felt that the ability to choose activities regardless of meaning, (e.g., activities that had no overt goal or direction) was an important part of participation. Similarly, when looking at the concept of participation in persons with disabilities, Martin Ginis et al. (11) identified themes related to choice and control, and to being valued and accepted by society. Martin Ginis et al. (11) also identified a theme related to “feeling appropriately challenged,” which was not overtly evident in our synthesis. Participants' desires to be treated equally and not be underestimated were, however, captured within our Being Included theme (47, 48, 50–52). Woodman et al. (10) also identified a theme of “pursuing personal choice,” which captured elements of our Self-Directed theme (10).

Elements of our theme, Do or Don't Do Mentality, were also reflected in Woodman et al. (10), which described references participants made about beliefs that they would persevere, or “beat” their injury (10). Whereas, some literature characterizes similar concepts as avoidance and endurance, noting the negative impacts that fear avoidance and “pushing through” despite limitations may have on symptom management (58), Woodman et al. (10) largely characterizes these findings as part of a process of reacclimating and reassessing. Similarly to Woodman et al. (10), the theme of Do or Don't Do Mentality from our synthesis cannot be definitively characterized as adaptive or maladaptive. Instead, the theme is reflective of a mentality about participation that participants expressed, the manifestation of which could vary by participant. Elements related to our Adaptation theme were also present in Woodman et al. 's (10) themes of building individual confidence and pursuing personal choice. Additionally, though termed “Having a sense of importance by doing” in one paper (20) and “Meaning” in another (11), content related to our theme, Contribution, was also prominent.

Van de Velde et al. (20) also highlighted the way in which participation can serve to fill time for participants, and how it can promote a sense of achievement, as outlined in our Outcomes of Participation descriptive themes. Sense of Accomplishment was captured under a broader theme of mastery in Martini Ginis et al. (11), which also divided “engagement” from choice, while we found references to choice and control generally quantified engagement and meaning in relation to personal interest, e.g. choice. Though outcomes of participation were discussed within the context “evaluating personal meaning,” Woodman et al. (10) captured similar outcomes from the articles she synthesized to those in this synthesis, such as Sense of Belonging and Sense of Self.

Though framed under different themes, each of the studies noted the impact on and importance of social relationships in ways that aligned with our Social Interactions and Relationships descriptive theme (10, 11, 20). Van de Velde et al. (20) also emphasized the importance of social interaction in terms that aligned with our descriptive theme of Being Included, but stressed that participants also defined participation as something that exists in a personal, private, and non-social context. Though not prevalent in our synthesis, there were quotes and observations that referenced the importance of individual, non-social, and independent activities, and a similar emphasis by authors of one of our synthesis articles, Hammel et al. (48). We generally captured these quotes in other themes such as Self-Directed or Normative Function (50–52). Further exploration of how pwABI may differentiate between the meaningfulness and importance of individual vs. social activities could help to refine definitions of participation and participation goals.

Several articles have articulated the challenges of integrating the ICF conceptual framework of participation with the perspectives of stakeholders (10, 11, 24, 54). Challenges of integration are echoed in the findings of this paper as some key elements of participation identified by pwABI could not be easily linked to a single category in the ICF. Several of the descriptive themes identified in the synthesis could not be linked beyond the component level of the ICF, or had “unspecified”, “other specified”, or *main concepts* that were not included in the Core Sets. Other qualitative syntheses highlighted similar issues with relating nuances of their themes to the ICF (10, 24).

An exception to these critiques is the summation of stakeholder perspectives on participation done by Magasi et al. (9). This paper reports on a large qualitative data set, which includes a data set published in one of our synthesis articles (48). The key domains identified by Magasi et al. (9) aligned with d7 “Interpersonal interactions and relationships”, d8 “Major life areas”, and d9 “Community, social and civic life,” specifically in the realm of “Recreation and leisure”. Our paper also found that d7 “Interpersonal interactions and relationships” and d9 “Recreation and leisure” (47–52) were key areas under Where pwABI Participate, though they were not necessarily part of how people defined participation. Our results further paralleled Magasi et al.'s (9) findings in that, while work did matter to participants, it was referenced far less often as a site of participation than the former two domains.

Our study findings highlight a relationship between our descriptive themes, such as Self-Directed and Advocacy and the ICF codes d940 “Human rights” and d950 “Political life and citizenship.” These categories most frequently align with the ways in which people define participation or think about approaching participation in our synthesis. As noted in the results section, these categories are not part of the ICF Comprehensive Stroke and TBI Core Sets (40, 41), and therefore are not considered by the ICF to be “essential” to people's function in these populations (42). Our paper identifies the potential importance of these categories to this population, a finding which aligns with recent research done in the field of TBI (59) and expert perspectives in a research symposium related to participation measurement (16). Many of the components of participation identified in this synthesis represent themes that are both challenging to orient within measurement and standardized models, and critical to how pwABI identify the construct of participation.

## Limitations

There were several limitations to this scoping review and synthesis. First, given the small number of qualitative articles addressing how pwABI define or conceptualize participation, it was not possible to address different types of brain injury separately. Three articles consisted solely of stroke participants (49–51), while others were a mix of persons with TBI and stroke (52) and TBI, stroke, and “other” (47, 48). Though themes did not seem to vary notably across stroke and combination articles, it is possible that the themes here are less representative of people with TBI. There may be differences between the perspectives of persons with TBI and persons with stroke that we were not able to identify. The small number of articles must also be taken into account when considering the applicability of results to the diverse experiences of pwABI. As noted above, qualitative research and particularly synthesis, relies on interpretation (10, 11), which can impact the results. To address the potential sources of bias we included three researchers in the formation of codes and themes, followed standards of independently attributing text to themes, compared levels of agreement, and discussed descriptive and analytical themes as a group to reach a resolution. It is also worth acknowledging that the CASP, while considered credible, has been critiqued for its limitations in addressing theoretical consistency in articles (35, 36). We also acknowledge that while initial agreement of ICF linking was strong for more granular descriptive themes, such as those under Where pwABI Participate, themes that encompassed more abstract information and larger sections of the ICF model as a whole, such as those under Essential Elements of Participation, were more challenging to link and required more discussion to reach agreement. Further, challenges outlined in previous works surrounding ICF linking were present throughout the linking process (60). The inclusion criteria requiring English language articles also increased the likelihood that results were predominantly reflective of the values and populations of western countries, and therefore limits the potential scope of the findings to western cultural contexts. Future research should focus on expanding data examining how both ABI and TBI stakeholders define participation, and efforts should be made to address the paucity of TBI-specific literature in this area. Additionally, researchers should try to explore this question in racial, ethnic, and cultural groups underrepresented in current literature. Research such as that done by Magasi et al. (9), which compared the perspectives on participation across different stakeholder groups, could serve as a guide for exploring these issues in neurotrauma.

## Conclusion

In this paper, we synthesized qualitative literature that reflects how participation is defined by pwABI and linked the resulting themes to the ICF. Our objective was to lay a foundation for better understanding how pwABI define participation, and how research and clinical fields can further identify and address stakeholder perspectives. We identified themes that illustrate how pwABI discuss participation in terms of its essential elements, their approach to how they participate, the domains in which participation is most important, and common outcomes of participation. Our results provide insight into the complexity of perspectives on participation among pwABI and illustrate aspects of participation that should hold elevated importance to clinicians and researchers as they try to support participation of pwABI.

## Data Availability Statement

The raw data supporting the conclusions of this article will be made available by the authors, without undue reservation.

## Author Contributions

CR, EE, CW, MS, and LK contributed to conceptualization of the project. CR, CW, and EE contributed to methodological development and analysis. CR and CW contributed to writing of the original manuscript. EE contributed to redrafting of the first manuscript. MB, LK, MS, EE, KE, CW, and SB contributed critical revisions of the work. All authors contributed to the article and approved the submitted version.

## Funding

This review was funded by the National Institute on Disability, Independent Living, and Rehabilitation Research 90DPCP0008.

## Conflict of Interest

The authors declare that the research was conducted in the absence of any commercial or financial relationships that could be construed as a potential conflict of interest.

## Publisher's Note

All claims expressed in this article are solely those of the authors and do not necessarily represent those of their affiliated organizations, or those of the publisher, the editors and the reviewers. Any product that may be evaluated in this article, or claim that may be made by its manufacturer, is not guaranteed or endorsed by the publisher.
