# Supplementary material for: Conceptualization of Participation: A Qualitative Synthesis of Brain Injury Stakeholder Perspectives
Source: Front Rehabil Sci. 2022 Jul 22;3:908615. doi: 10.3389/fresc.2022.908615 (PMC9397755; doi:10.3389/fresc.2022.908615)
Supplement: Supplementary file 1 [file Table_1.DOCX]

Supplementary Material

Supplemental Table 1. Examples of article quotes that support each theme.

| Analytical Theme | Descriptive Theme | Examples of Quotes |
| --- | --- | --- |
| Essential Elements of Participation | Being Included | “It means taking part, being one of the team...a cog within a wheel” The stroke survivors described how participation meant being part of something, which involved working in co-operation with others.” (51) |
|  | Beyond Function | “Participation can be partial, from the perspective of society, but full and meaningful in the eyes of the person it involves.” (47) |
|  | Normative Function | “Being able to do favourite activities alone which had always been done alone, was described as a form of participation…” (51) |
|  | Contribution | “In resistance to popular perceptions about people with disabilities as perpetually receiving help, the root of participation for many participants was defined not by what they can get from other people, but instead by what they can contribute back to others.” (48) |
|  | Self-Directed | “For example, participants defined participation in life as: Just to be able to do whatever you want to do to your fullest extent to the best of your ability.” (48) |
| How pwABI Approach Participation | Adapted Tasks | “This is a one handed knitting holder. Someone in the group suggested I try it out so I would be able to knit. It’s a good thing that I can use it.” (50) |
|  | Unadapted Tasks | “Let's say I'm watching soaps...Discovery Channel...! turn the channel, or watch a different programme because I can't remember the stories. Because the memory is not there. And likewise too for reading, I don't read much. Because I can't remember.” (49) |
|  | Reassignment of Value | “The informants also revalued the “few” things they did for others because these things and the people concerned had become much more significant to them, making these activities more important for their sense of participation than they had been before.” (52) |
|  | Advocacy | “We have a unique insight into life that a lot of people don’t have. I think that’s really important when we’re talking about participation that we can share with others that haven’t experienced this . . . from our perspective.” (48) |
|  | Do or Don’t Do Mentality | “You can actually do more, than you thought you would dare, so you just have to do it.” (47) |
| Where pwABI Participate | Social Interactions & Relationships | “The informants’ experiences reflected how their participation was enhanced by prioritization of activities conducted with those who made them feel good, such as people close to them, persons who had their own experiences of having a disability and pets.” (52) |
|  | Leisure | “Well after I had my stroke, it was about 2 month after I went back to the [gym].” (50) |
|  | Work | “I think maybe for me it would be working . . . I got a real high need to be doing something useful. I have been forced into early retirement by the fact that if I do go back to work I could lose the benefits that I have right now.” (48) |
|  | Volunteer | “For example, Andy who had aphasia following his stroke shared a picture of the place that he had recently begun volunteering. He talked about it as follows: ‘‘Go here once a week. It’s exciting.” (50) |
|  | Education | “I do art classes.” (49) |
|  | Health Management | “Pain killers. It’s just constant, how much you take. It’s trying not to taking it. There is endless cautions to not taking. It helps, but how much can you afford to take.” (50) |
|  | Community | “All participants had become members of stroke support groups and/or stroke clubs.” (49) |
|  | Assisting People | “Sometimes I help people… they call me to ask [for help]… and then I feel that I am appreciated and… participating.” (52) |
|  | Domestic Tasks | “*I tried to pick-up my old life:*…*and doing some activities regarding our home.” (47) |
|  | Religion | “Another important and deeply personal form of connection was expressed in people’s religious or spiritual faith. Spirituality and faith were identified as an important source of strength, comfort and motivation.” (48) |
|  | Financial Independence | “*I want to have access** to….buy a home….” (48) |
|  | Communication | “People often have problems understanding me, but they pretend to understand me. Maybe that’s the hardest part of it!” (47) |
|  | Physical Function | “Walking is difficult and I was trying to use this to represent the problems…I used to walk up and down the block all the time with my wife. But it’s just too hard now. I’m too slow.” (50) |
|  | Use of Transportation | “With respect to accessibility, participants spoke of their inability to use regular public transportation owing to the difficulty they encountered while trying to enter the buses.” (49) |
| Outcomes of Participation | Fills Time | “Say if you did crosswords or Sudoku and that takes away hours of your time. I mean that’s how I spend my time…it keeps me active mentally.” (50) |
|  | Sense of Accomplishment | “I can still do the things that I did, maybe in a different way, but I can still accomplish this or that.” (48) |
|  | Sense of Belonging | “Other stroke survivors described the benefits of socializing, such as maintaining old and developing new friendships, being able to relate to others with similar experiences and the enjoyment of having companionship while participating in their leisure activities.” (49) |
|  | Sense of Satisfaction | “Doing these tasks, taking care for others, gives me a sense of worth and satisfaction.” (47) |
|  | Sense of Self | “It was apparent that participation had a profound meaning for stroke survivors, that of defining who they are. “Doing” and “being” were often mentioned simultaneously, suggesting that they are intertwined.” (51) |
|  |  |  |

*Italicized text was not originally coded under descriptive themes but was added for context.
